# Occurrence and molecular phylogeny of *Fasciola* species in camels of southwestern Iraq

**DOI:** 10.14202/vetworld.2024.2957-2966

**Published:** 2024-12-26

**Authors:** Isra’a M. Essa, Ghazi Y. Azzal

**Affiliations:** Department of Public Health, College of Veterinary Medicine, University of Basrah, Basra, Iraq

**Keywords:** camel trematode, conventional polymerase chain reaction, fascioliasis, one-humped camel, phylogenetic analysis

## Abstract

**Background and Aim:**

*Fasciola* spp. are important trematodes of public health concern in various animals, including camels. This study aimed to determine the occurrence of liver flukes in camels, to determine the molecular confirmation of *Fasciola*, and to perform phylogenetic analysis of study isolates to identify the species of *Fasciola*.

**Materials and Methods:**

In total, 107 slaughtered camels were inspected to collect liver flukes that were examined molecularly using polymerase chain reaction (PCR) to confirm *Fasciola* species. Then, the study isolates were sequenced, submitted to the National Center for Biotechnology Information (NCBI) database, and analyzed phylogenetically to identify the species of each study isolate.

**Results:**

Liver flukes were detected in 17.67% of the camels. Regarding the migratory stages of the collected worms, juvenile worms (73.91%) were significantly more prevalent than adult worms (26.09%). Regarding the risk factors, a significantly greater occurrence rate and risk of infection was detected in Al-Najaf compared with Al-Muthanna, as well as in younger camels (1–4 years) compared with older camels. Although the occurrence rate of liver flukes was insignificantly different between females (9.38%) and males (6.98%), females appeared to be at a significantly higher risk of infection than males. Molecularly, 33.33% of the worm samples were positive for species in the *Fasciola* genus. Phylogenetic analysis of all positive PCR products (total no = 19) confirmed that 63.16% of the local *Fasciola* spp. isolates were related to the NCBI-Basic Local Alignment Search Tool (NCBI-BLAST) Saudi Arabian *Fasciola hepatica* isolate at an identity range of 95.94%–99%; while 36.84% of the local *Fasciola* spp. isolates were related to the NCBI-BLAST Iranian *F. gigantica* isolate at an identity range of 97.73%–99%.

**Conclusion:**

This study found a 17.67% occurrence of *Fasciola* spp. in camels, with juvenile worms being more common than adult worms. Molecular analysis revealed that 63.16% of the isolates were related to *F. hepatica* from Saudi Arabia, while 36.84% matched *F. gigantica* from Iran. Younger camels and those from Al-Najaf were at higher risk, highlighting the need for targeted control measures.

## Introduction

*Fasciola*, which is commonly known as liver fluke, is a genus of trematode parasites that have been described as dorsoventrally flattened, bilaterally unsegmented, and soft-bodied invertebrates that cause a disease known as fasciolosis or fascioliasis [1]. Taxonomically, although various species are found within the genus *Fasciola*, *F. hepatica* and *F. gigantic* alone are well known [2]. These parasites have economic and welfare importance in the livestock sector, particularly in cattle, camels, sheep, and goats, because of changes in climate and farming practices [3]. *Fasciola* has a complete life cycle, including free-living stages and an intermediate host (snails). Adult parasites reside in the large bile ducts of the host, where they shed eggs into the gall bladder. Eggs are transported along the intestine to be excreted in the host’s feces [4, 5]. In the environment, once eggs are freed from fecal matter, free-swimming miracidia hatch and penetrate a suitable snail intermediate host, then migrate to the digestive duct of snail where they transform into sporocysts. After many asexual reproduction rounds, large numbers of cercariae are produced and released from the snail host, followed by migration to vegetation and the formation of resistant cysts or metacercaria, which are ingested by mammalian hosts. Finally, metacercaria excyst in duodenum, migrate to the liver as immature flukes, and then develop into adult individuals in the bile ducts [6, 7].

The dromedary camel (*Camelus dromedaries*) is an even-toed, ungulate animal that has adapted physiologically and anatomically to survive the harsh conditions of desert and semi-desert areas in numerous countries located in Asia, Africa, and South America [8]. In Iraq, the number of camels was estimated at 330,000 in the 1970s and had decreased to 65,000 in 2014 [9]; recently, unofficial data are describing increasing numbers of camels in Iraq, up to 205,000, distributed throughout different areas, particularly in Al-Anbar, Al-Najaf, Al-Muthanna, and Nineveh. Like domestic animals, camels are susceptible to various infectious and pathogenic agents [2, 10]. Enteric infections in camels caused by a number of helminths are rare because of their typical browsing habits; however, several previous [11–13] and recent [14–16] studies have indicated an increase in the incidence of fasciolosis in camels.

Because of the chronic nature of *Fasciola* infection in camels, a confirmatory diagnosis can be performed traditionally via the direct identification of eggs and/or adults in feces using coprological and coproantigen techniques or indirectly by detecting specific anti-*Fasciola* antibodies using serological assays. However, each approach has drawbacks, as they require a high level of experience, considerable effort, and time [17]. In recent years, the continuous development of molecular technologies for the rapid estimation of the prevalence of different infections has been achieved at a high level of sensitivity and specificity, with a shorter detection time and increased automation [18]. Phylogenetic analyses are additional advanced tools that have allowed the study of the evolutionary development of a species or group of organisms and gathering information on biological diversity [19, 20]. In Iraq, camel fasciolosis is conventionally diagnosed [21–23].

Therefore, the present study was designed to determine the occurrence of liver flukes in the liver and bile ducts of slaughtered camels, followed by the molecular and phylogenetic confirmation of *Fasciola* isolates for the first time in Iraq.

## Materials and Methods

### Ethical approval

This study was approved by the Scientific Committee of the Department of Parasitology, College of Veterinary Medicine, University of Basrah, Basra, Iraq (DP-CVM-2/17-10-2023). All animal procedures were conducted in accordance with the Declaration of Helsinki on animal welfare.

### Study period and location

The study was conducted from October 2023 to March 2024. The samples were collected from Al-Muthanna and Al-Najaf official and private slaughterhouses. The samples were processed at the Department of Parasitology, College of Veterinary Medicine, University of Basrah.

### Samples

**Figure-1:**
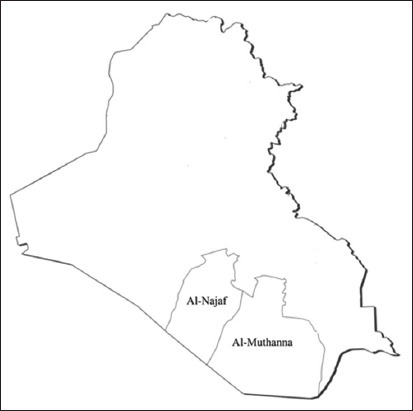
Geographic location of the study areas. Adobe Photoshop Software, version 21 (Adobe Inc., USA) was used to preparing the map.

In this study, 107 slaughtered camels were subjected to post-slaughter inspection (Figure-1) to detect worms in hepatic tissues and bile ducts. All detected worms were collected in labeled plastic Petri dishes containing phosphate-buffered saline (pH ~7.4). In the laboratory, the worms were washed thoroughly with PBS, fixed in 70% ethanol, and frozen (–4°C) in Eppendorf tubes until they were tested molecularly using a conventional polymerase chain reaction (PCR) assay.

### Molecular assay

According to the Type B Protocol of the G-Spin Total DNA Extraction Kit (iNtRON Biotechnology, Korea), DNA was extracted and tested using a Nanodrop System (Thermo Scientific, UK) to measure the concentration and purity at a range of 50–150 μg/mL and 1.8–2.2, respectively. The MasterMix tubes were prepared at a final volume of 25 μL (5 μL DNA template, 2 μL forward primer, 2 μL reveres primer, and 16 μL nuclease-free water) using the GoTaq^®^ G2 Master Mixes Kit (Promega, Korea) and one set of primers ([IMF: 5′–AGTAACGGCGAGTGAACAGG–3′] and [IMR: 5′–ACAAGCAGGTCCGTCAGTAC–3′]) targeting the *18S rRNA* gene based on the National Center for Biotechnology Information (NCBI)-GenBank *Fasciola* spp. isolate (OR676767.1). PCR amplification was conducted on a T100 Thermal Cycler System (Bio-Rad, USA) under the following modified conditions: 1 cycle of initial denaturation (95°C for 5 min); followed by 35 cycles of denaturation (95°C for 30 s), annealing (55°C for 30 s), and extension (72°C for 2 min); and a final cycle of extension (72°C for 5 min). Electrophoresis was performed at 100 V and 80 Am for 90 min on 1.5% agarose gels, which were then stained with ethidium bromide. In parallel with the bands of the ladder marker (iNtRON Biotechnology, Korea), the PCR products were measured under an ultraviolet transilluminator (ATTA, Korea); the expected product size of the positive samples was 534 bp.

### Phylogenetic analysis

The DNA of all positive samples was sequenced by Macrogen Company, Ltd. (South Korea) using the Sanger sequencing method. To detect their species, the sequence data of the study isolates were subjected to a phylogenetic tree analysis using the NCBI-Basic Local Alignment Search Tool (NCBI-BLAST). Then, the *Fasciola* isolates were submitted to the NCBI-GenBank with following accession numbers; PQ226379.1, PQ226380.1, PQ226381.1, PQ226382.1, PQ226383.1, PQ226384.1, PQ226385.1, PQ226386.1, PQ226387.1, PQ226388.1, PQ226389.1, and PQ226390.1 for *F. hepatica* isolates, and PQ237736.1, PQ237737.1, PQ237738.1, PQ237739.1, PQ237740.1, PQ237741.1, and PQ237742.1 for *F. gigantica* isolates.

### Statistical analysis

Initially, all collected data were recorded in Microsoft Office Excel, version 2010 (Microsoft, Washington, USA) and then tested using a t-test to determine the relative risk (RR) and odds ratios (OR) in the MedCalc Software (MedCalc Software Ltd, Belgium). Values are presented as the mean ± standard error, and differences were considered significant at p < 0.05 [24]. The figures were prepared using GraphPad Prism version 9.0.1 (GraphPad Software Inc., USA).

## Results

**Figure-2:**
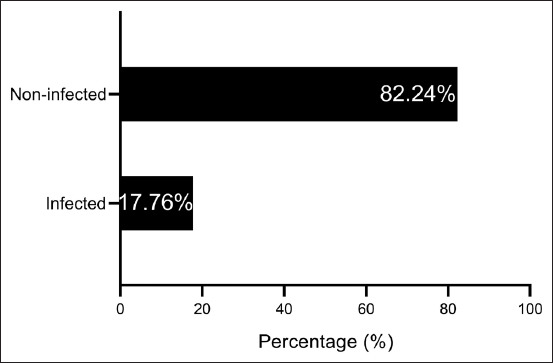
Results of gross inspection of liver organs collected from 107 slaughtered camels.

**Figure-3:**
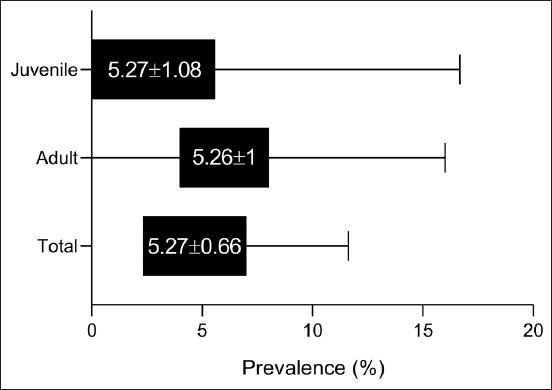
Total number of collected worms and their classification into adult and juvenile liver flukes according to migratory stage.

A gross inspection of the liver organs in the 107 slaughtered camels revealed that 19 (17.76%) livers were infected with worms at different migratory stages and numbers (Figure-2). The total number of collected worms was 43, including 25 (58.14%) adults and 18 (41.86%) juvenile worms (Figure-3). The highest and lowest numbers of collected worms from each camel were 5 (21.74%) and 1 (4.35%), respectively (Table-1).

**Table-1:** Classification of the worms collected from each infected camel according to its migratory stage.

No. of sample	Worm (%)		

	Total	Adult	Juvenile
7	3 (6.98)	2 (8)	1 (5.56)
11	1 (2.33)	0 (0)	1 (5.56)
18	4 (9.3)	1 (4)	3 (16.67)
21	2 (4.65)	0 (0)	2 (11.11)
36	5 (11.63)	4 (16)	1 (5.56)
39	3 (6.98)	2 (8)	1 (5.56)
44	3 (6.98)	1 (4)	2 (11.11)
46	1 (2.33)	1 (4)	0 (0)
47	1 (2.33)	0 (0)	1 (5.56)
53	1 (2.33)	1 (4)	0 (0)
59	3 (6.98)	1 (4)	2 (11.11)
65	1 (2.33)	1 (4)	0 (0)
68	1 (2.33)	0 (0)	1 (5.56)
71	1 (2.33)	1 (4)	0 (0)
74	2 (4.65)	2 (8)	0 (0)
91	4 (9.3)	3 (12)	1 (5.56)
93	2 (4.65)	1 (4)	1 (5.56)
97	3 (6.98)	2 (8)	1 (5.56)
105	2 (4.65)	2 (8)	0 (0)
p-value	p < 0.0001	p < 0.0001	p < 0.001
Confidence interval	3.876–6.655	3.226–7.301	2.994–7.536

Regarding the risk factors, a significant variation was observed between the groups for each factor (Table-2). The occurrence rates of worms did not differ significantly (p > 0.05) between Al-Muthanna (16.13%) and Al-Najaf (18.13%). However, the OR and RR values were significantly (p < 0.0001) higher in camels from Al-Najaf (1.1742 and 1.1200, respectively) than in camels from Al-Muthanna (0.8516 and 0.8929, respectively).

**Table-2:** Association between infected camels (n = 19) and risk factors.

Factor (group)	Total	Positive no. (%)	Odds ratio		Relative risk		
	
			Value	95% CI	Value	NNT	95% CI
Region							
Al-Muthanna	31	5 (16.13)	0.8516	0.2781–2.6079	0.8929	60 (Benefit)	0.347–2.2975
Al-Najaf	76	14 (18.42)	1.1742	0.3835–3.5956	1.1200	60 (Harm)	0.4353–2.8820
Sex							
Female	43	8 (18.61)	1.1013	0.4028–3.0111	1.0695	98.077 (Harm)	0.4625–2.4734
Male	64	11 (17.19)	0.9080	0.3321–2.4827	0.9350	98.077 (Benefit)	0.4043–2.1623
Age (year)							
<1	19	1 (5.26)	0.2160	0.0270–1.7281	0.2944	8.346 (Benefit)	0.0416–2.0824
1–4	41	13 (31.71)	4.6429	1.5984–13.4857	2.8889	6.353 (Harm)	1.1737–7.1106
>4–8	35	4 (11.43)	0.4903	0.1497–1.6061	0.5949	14.316 (Benefit)	0.2110–1.6767
>8	12	1 (8.33)	0.3889	0.0471–3.2092	0.4829	12.140 (Benefit)	0.0701–3.3275

CI=Confidence interval, NNT=Number needed to treat

Regarding the sex of the camels, although there was an insignificant variation (p > 0.05) in the prevalence rate of infection between females (18.61%) and males (17.19%), the OR and RR values significantly indicated that females (1.1013 and 1.0695, respectively) were at higher risk of infection compared with males (0.9080 and 0.9350, respectively).

Regarding the different age groups, the positivity, OR, and RR values were increased significantly in camels aged 1–4 years (31.71%; 4.6429 and 2.8889, respectively) and decreased significantly in camels aged <1 year (5.26%; 0.2160 and 0.2944, respectively) compared with those aged >4–8 (11.43%; 0.4903 and 0.5949, respectively) and >8 (8.33%; 0.3889 and 0.4829, respectively) years.

**Figure-4:**
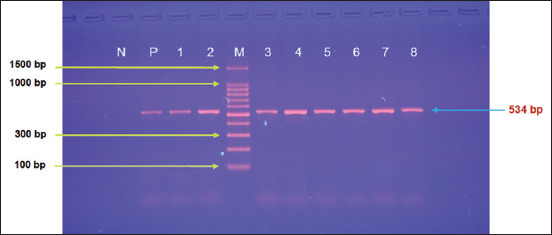
Representative image of polymerase chain reaction products analyzed using agarose gel electrophoresis at 100 V and 80 Am for 1 h. Lane (M)=Ladder marker (100–1500 bp); Lane (N)=Negative control; Lane (P)=Positive control; Lanes (1–8)=Positive Fasciola spp. at 534 bp.

**Figure-5:**
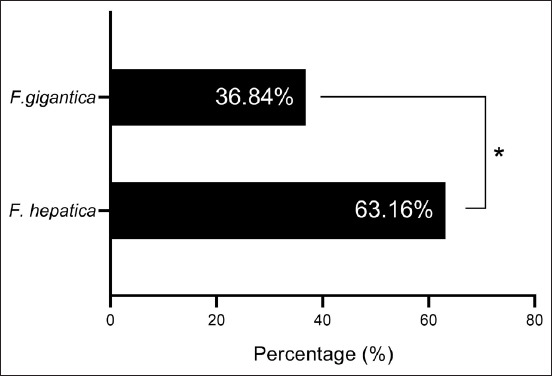
Prevalence of *Fasciola hepatica* and *Fasciola gigantica* in camels based on phylogenetic analysis.

**Figure-6:**
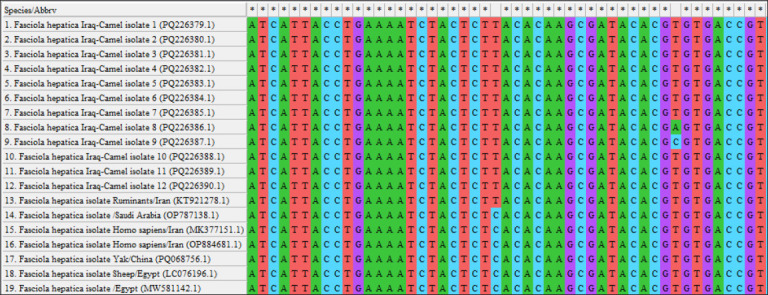
Multiple sequence alignment of local and National Center for Biotechnology Information-Basic Local Alignment Search Tool *Fasciola hepatica* isolates.

**Figure-7:**
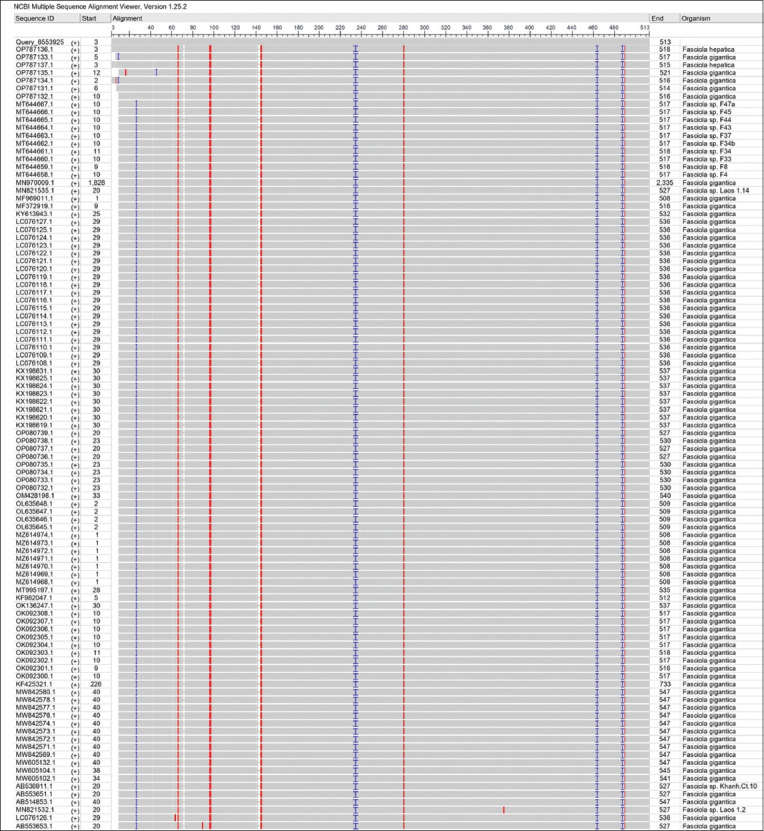
Multiple sequence alignment analysis of local and National Center for Biotechnology Information-Basic Local Alignment Search Tool *Fasciola hepatica* isolates using the NCBI-MSA Viewer.

**Figure-8:**
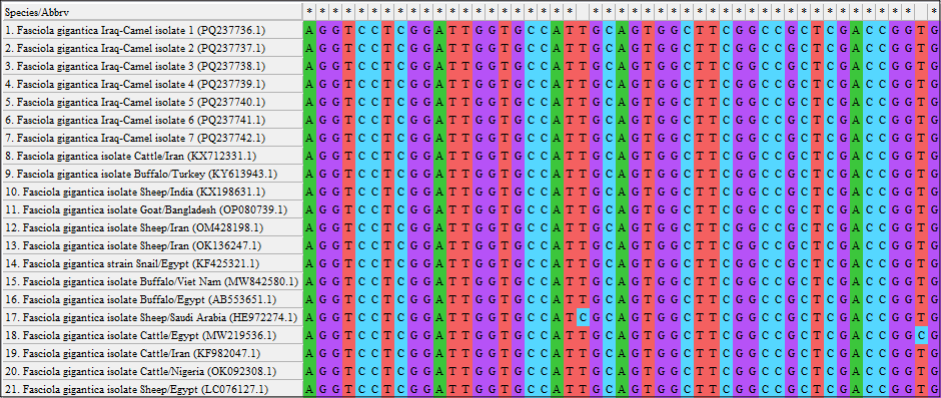
Phylogenetic tree analysis of local and National Center for Biotechnology Information-Basic Local Alignment Search Tool *Fasciola gigantica* isolates.

**Figure-9:**
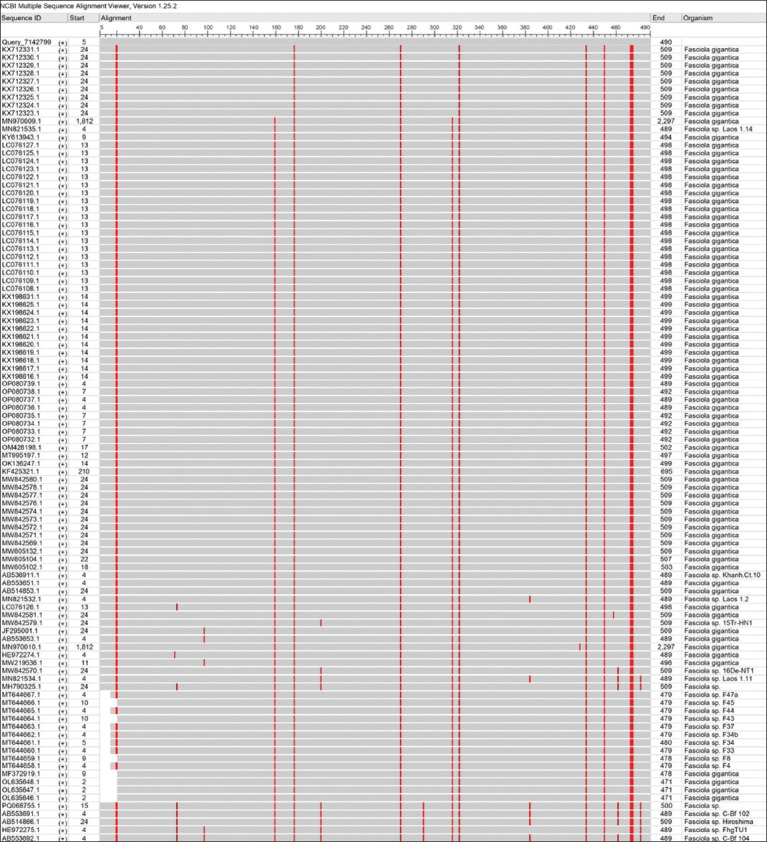
Multiple sequence alignment of local and National Center for Biotechnology Information-Basic Local Alignment Search Tool *Fasciola gigantica* isolates using the NCBI-MSA Viewer.

**Figure-10:**
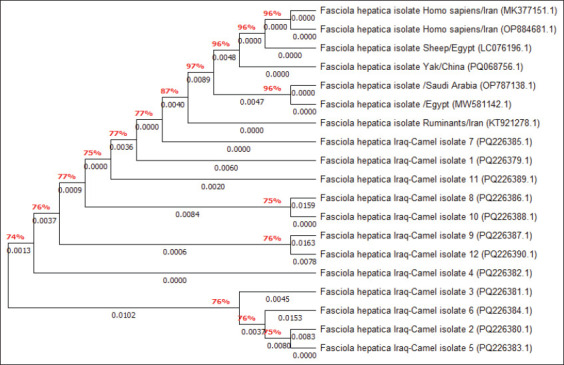
Phylogenetic tree analysis of local and National Center for Biotechnology Information-Basic Local Alignment Search Tool *Fasciola hepatica* isolates.

**Figure-11:**
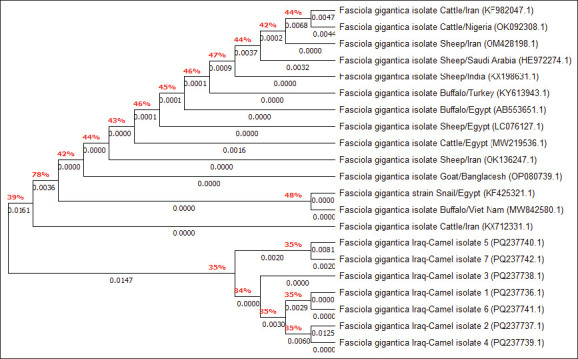
Phylogenetic tree analysis of local and National Center for Biotechnology Information-Basic Local Alignment Search Tool *Fasciola gigantica* isolates.

By targeting the *18S rRNA* gene, the molecular examination of the camel worms using a PCR assay revealed that all samples were positive for *Fasciola* spp. (Figure-4). Subsequently, phylogenetic tree analysis of all positive PCR products confirmed that 12 (63.16%) of the local *Fasciola* spp. isolates were related to the NCBI-BLAST Saudi Arabian *F. hepatica* isolate at an identity range of 95.94%–99%; while 7 (36.84%) of the local *Fasciola* spp. isolates were related to the NCBI-BLAST Iranian *F. gigantica* isolate at an identity range of 97.73%–99% (Figure-5, Tables-3 and 4). Compared with the different NCBI-BLAST isolates, the multiple sequence alignment and phylogenetic tree analysis data indicated different degrees of similarity (*) and mutation/changes (Figures-6–11).

**Table-3:** Homology sequence identity (%) between local *Fasciola* spp. isolates and NCBI-BLAST *F. hepatica* isolates.

Local isolate		NCBI isolate			
	
Name	Accession no.	Species	Country	Accession no.	Identity (%)
Iraq-Camel 1	PQ226379.1	*F. hepatica*	Saudi Arabia	OP787136.1	99
Iraq-Camel 2	PQ226380.1	*F. hepatica*	Saudi Arabia	OP787136.1	98.96
Iraq-Camel 3	PQ226381.1	*F. hepatica*	Saudi Arabia	OP787136.1	96.57
Iraq-Camel 4	PQ226382.1	*F. hepatica*	Saudi Arabia	OP787136.1	96.45
Iraq-Camel 5	PQ226383.1	*F. hepatica*	Saudi Arabia	OP787136.1	95.94
Iraq-Camel 6	PQ226384.1	*F. hepatica*	Saudi Arabia	OP787136.1	96.33
Iraq-Camel 7	PQ226385.1	*F. hepatica*	Saudi Arabia	OP787136.1	99
Iraq-Camel 8	PQ226386.1	*F. hepatica*	Saudi Arabia	OP787136.1	98.55
Iraq-Camel 9	PQ226387.1	*F. hepatica*	Saudi Arabia	OP787136.1	95.54
Iraq-Camel 10	PQ226388.1	*F. hepatica*	Saudi Arabia	OP787136.1	97.63
Iraq-Camel 11	PQ226389.1	*F. hepatica*	Saudi Arabia	OP787136.1	98.87
Iraq-Camel 12	PQ226390.1	*F. hepatica*	Saudi Arabia	OP787136.1	97.06

NCBI-BLAST=National Center for Biotechnology Information-Basic Local Alignment Search Tool, *F. hepatica*=*Fasciola hepatica*

**Table-4:** Homology sequence identity (%) between local Fasciola spp. isolates and NCBI-BLAST *F. gigantica* isolates.

Local isolate		NCBI isolate			
	
Name	Accession no.	Species	Country	Accession no.	Identity (%)
Iraq-Camel 1	PQ237736.1	*F. gigantica*	Iran	KX712331.1	97.94
Iraq-Camel 2	PQ237737.1	*F. gigantica*	Iran	KX712331.1	98.50
Iraq-Camel 3	PQ237738.1	*F. gigantica*	Iran	KX712331.1	99
Iraq-Camel 4	PQ237739.1	*F. gigantica*	Iran	KX712331.1	97.93
Iraq-Camel 5	PQ237740.1	*F. gigantica*	Iran	KX712331.1	97.73
Iraq-Camel 6	PQ237741.1	*F. gigantica*	Iran	KX712331.1	98.94
Iraq-Camel 7	PQ237742.1	*F. gigantica*	Iran	KX712331.1	97.94

NCBI-BLAST=National Center for Biotechnology Information-Basic Local Alignment Search Tool, *F. gigantica*=*Fasciola gigantica*

## Discussion

Intestinal parasites are major obstacles to health and growth of animals. In areas located around canals, trematodes such as *Fasciola* spp. are of great importance, as they lead to severe economic losses not only by reducing the productivity and performance of camels but also by predisposing them to other infections [25, 26]. After a gross inspection of the livers and bile ducts of slaughtered camels, our findings revealed that 8.41% of the camels were infected with these worms. In Iran, the livers of 409 slaughtered camels showed that 5.3% of the animals harbored *F. hepatica* flukes, with an average of 10.5 parasites per animal [27]. Another study by Yazdanbakhsh *et al*. [28] examined the carcasses of 94 camels and reported only one infected camel (1.06%). In Mogadishu (Somalia), coprological examination of fecal samples from 167 camels revealed that the overall prevalence of *Fasciola* spp. was 0.6%, regardless of their clinical status [29].

In Iraq, Karawan [25] aimed to estimate the prevalence rate of intestinal parasites in camels by examining 110 fecal samples, and the findings revealed that *Fasciola* spp. exhibited a maximum infection rate (31%). Wakil *et al*. [30] reported that the prevalence of *Fasciola* spp. in fecal samples from 202 camels was 0.9%. In Algeria, microscopic examination of 717 fresh fecal samples obtained from 28 camel farms in the Sahara region [14] and 100 fecal samples collected from dromedaries in the Laghouat region [31] indicated that the occurrence of *Fasciola* spp. were 0.42% and 4%, respectively. In Egypt, El-Dakhly *et al*. [15] and El-Khabaz *et al*. [32] performed coprological detections of *Fasciola* spp. in 120 clinically suspected camels and 626 domestic dromedaries and reported prevalences of 3.3% and 1.12%, respectively. Bekele *et al*. [33] reported the prevalence of *Fasciola* spp. from 384 camels at 4.9% in Ethiopia. In contrast, other findings reported a lack of *Fasciola* infection during the examination of the livers of slaughtered camels in Jeddah, Saudi Arabia [34] and Kazerum and Shiraz abattoirs, Iran [35]. The discrepancies observed between the findings of the different studies might be attributed to the diagnostic method used, the time of sampling, and the number of animals screened.

According to the results pertaining to the risk factors, no significant difference was observed in the occurrence of liver flukes among the study regions, which is in agreement with the findings of previous studies by Ibrahim *et al*. [29] and Bekele *et al*. [33]. However, variations may have occurred because of the type of agricultural and production systems, the presence of other domestic animals (such as goats, cattle, and sheep), climatic conditions, and husbandry practices, as well as the topographical nature of the study regions.

Regarding the sex of the animals, no significant variation was observed in the infection rate among female and male camels; however, females appeared to have a higher infection rate than males. These findings are similar to those of other studies on camels [14, 29, 31, 34]; however, these studies are in contrast with Wakil *et al*. [30], who identified an increasing prevalence of fasciolosis in females versus males. We suggest that the increased risk of infection observed in females can be attributed to the increased stress experienced by female camels due to pregnancy, parturition, seasonal variation in reproductive hormones, and milk production. Regarding the age of the camels under study, we found that young camels (aged 1–4 years) had a higher rate of infection compared with those in the other age groups, as reported by Bouragba *et al*. [14], but in contrast with results of other studies that found no significant association between age and infection [30, 31, 33]. The attenuation of maternal immunity and increased exposure to the parasite may be the main causes of the increased disease incidence among young animals.

Molecular techniques have become widely accepted worldwide because they provide more specific and sensitive information than traditional methods [36]. By targeting the *18S rRNA* gene in the present study, conventional PCR analysis confirmed that all collected liver flukes belonged to the *Fasciola* genus. Compared with the NCBI-BLAST isolates, the phylogenetic analysis performed here confirmed that 63.16% of the study isolates exhibited significant identity to *F. hepatica*; in contrast, 36.84% were identical to *F. gigantica* isolates. Worldwide, DNA amplification using molecular assays has been used to support the taxonomy of various parasites using mitochondrial DNA markers, such as cytochrome oxidase subunit I and NAD1, and/or ribosomal DNA markers, including internal transcribed spacer genes (ITS1 and ITS2) [37–42]. The *18S rRNA* gene is well-suited to the investigation of the genetics of trematodes [43–45]. In turn, mitochondrial markers have been extensively used to conduct phylogenetic studies and distinguish different pathogen species because of their relativity rapid rate of evolution, importance in the differentiation and discrimination of closely related organisms, maternal inheritance, and lack of recombination [46].

## Conclusion

This is the first study in Iraq which highlights the significant occurrence of *Fasciola* spp. liver flukes in camels, with a rate of 17.67%. Juvenile worms were notably more prevalent than adult worms, suggesting active or recent infections. Regional differences revealed a higher risk of infection in camels from Al-Najaf compared to Al-Muthanna. Younger camels (1–4 years) were more susceptible to infection, and while the occurrence rate between males and females was not significantly different, females appeared at a higher risk of infection.

Molecular analysis confirmed the presence of *Fasciola* species, with 63.16% of isolates closely related to *F. hepatica* from Saudi Arabia and 36.84% matching *F. gigantica* from Iran. These findings provide valuable insights into the epidemiology of liver flukes in camels, emphasizing the need for region-specific control measures to mitigate the risk of infection and potential public health implications. The population genetics of *Fasciola* parasites in Iraq needs to be studied extensively, as intraspecific genetic variations have been observed among liver flukes, which may reflect apparent differences in virulence, host specificity, and drug susceptibility or resistance.

## Data Availability

All data generated during the study are included in the manuscript.

## Authors’ Contributions

IME: Collection and molecular examination of worm samples and drafted and revised the manuscript. GYA: Designed the study, statistical analysis, and phylogenetic analysis of local *Fasciola* isolates. Both authors have read and approved the final manuscript.
